# Accelerated Water Transportation Phenomenon through a Hydrophilic Metal Roll

**DOI:** 10.1021/acsaenm.3c00468

**Published:** 2023-10-17

**Authors:** Xiaojie Liu, Xuguang Zhang, Fangqi Chen, Yanpei Tian, Ying Mu, Marilyn L. Minus, Yi Zheng

**Affiliations:** † Department of Mechanical and Industrial Engineering, Northeastern University, Boston, Massachusetts 02115, United States; ‡ Department of Chemical Engineering, Northeastern University, Boston, Massachusetts 02115, United States

**Keywords:** water evaporation, water transportation, copper oxide nanostructures, hydrophilicity, metal roll

## Abstract

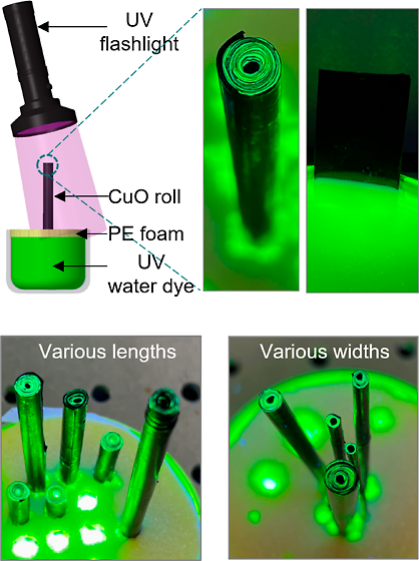

Passive water transport by taking advantage of capillary forces is vital for various applications such as solar-driven interfacial evaporation, evaporative cooling, and atmospheric water harvesting. Surface engineering and structure design with a hydrophilic surface and enhanced capillary force will facilitate passive water transport. Herein, we demonstrate a hydrophilic Cu/CuO foil-based roll for accelerated water transportation. The roll was fabricated by rolling up a typical 2D Cu/CuO film, which transforms the water climbing behavior by significantly enhancing the capillary force between each Cu/CuO film layer. The simple spatial transformation for a 2D film, from planar foil to 3D structure, has extensively facilitated water transportation performance and broadened its practical application potential. The Cu/CuO film with a blade-like nanostructure and excellent hydrophilicity ensures water supply to a limited area, while the capillary effect between different layers of the Cu/CuO foil extends the water transportation height. Consequently, the Cu/CuO foil-based roll demonstrated a high fluidic transport velocity. This design derived from the 2D planar film can be potentially employed for a large range of applications such as evaporating in a confined space and evaporation-driven energy harvest.

## Introduction

The recent increase in concern about water scarcity and severe pollution of natural water bodies necessitates efficient and cost-effective water purification techniques.^1−5^ Moreover, the increasing power density and reduced sizes of electronic devices of 5G stations and data centers necessitate evaporative cooling to facilitate fast thermal dissipation.^6−13^ In a solar-driven interfacial evaporator, artificially designed or naturally existing channels are employed to pump efficient water to the evaporation interface and protect evaporators from salt accumulation, allowing for continuous water desalination. In the evaporative cooling device for compacted electronic devices, the premise for effective evaporative is sufficient water transportation to the evaporation surface. Accordingly, developing passive water transport techniques that can pump water without energy input is significant to enhance the water evaporation rate and cooling efficiency.^14,15^

The capillary phenomenon is the process of a liquid flowing in a narrow space without the assistance of, or even in opposition to, any external forces.^16−19^ Water adhesion to a vessel’s walls will exert upward strain on the liquid, causing a meniscus to turn upward. Surface integrity is maintained by surface tension. When the cohesive forces between the liquid molecules are greater than the adhesion to the walls, capillary action results. Water adhesion force is enhanced when the surface is in a hydrophilic state.^16^ Therefore, a structure with a narrow gap and a hydrophilic modified surface will substantially enhance the water transport performance.

Structured copper dioxide (CuO) surface grown by hot alkaline solution treatment of the copper (Cu) plate has been employed in different applications. Liu et al. demonstrated a single planar Cu foam with nanostructured copper oxide (CuO) on the surface and an interconnected open-pore structure (Cu/CuO foam) as an integrative 2.5D photothermal evaporator with excellent solar evaporation performance.^4^ Miljkovic et al. validated that jumping-droplet condensation heat transfer is extremely effective on surfaces made of silanized copper oxide using a straightforward manufacturing technique.^20^ However, their investigations are focused on how surface modification of CuO nanostructures affects the water transportation phenomenon. The water transport behavior between a narrow gap and a hydrophilic surface has not been investigated. Therefore, structuring the CuO-coated surface into narrow gaps that can pump water passively will enhance the improvement of solar evaporation and passive evaporative cooling.

In this work, we present a unique design for a hydrophilic Cu/CuO foil-based roll that speeds up water transit. The roll was created by rolling up a standard 2D Cu/CuO film. This changes the behavior of the water when climbing up the Cu/CuO film by considerably increasing the capillary force between each layer. A 2D film’s straightforward spatial translation from a planar foil to a 3D structure greatly improved water transportation performance and increased the range of possible practical applications. The narrow gap is formed after the partial release of the rolled 2D film. The capillary action between the various layers of Cu/CuO foil increases the height of water transportation, while the Cu/CuO film’s blade-like nanostructure and superior hydrophilicity provide water delivery to a specific location. This results in a Cu/CuO foil-based roll exhibiting a high fluidic transport velocity. Enhanced water transportation is crucial for solar-driven water evaporation, desalination, and cooling applications. A wider range of applications, including evaporating sterilant to kill airborne germs and viruses in a small space and evaporation-driven energy harvest, are possible with this design evolved from the 2D planar film.

## Experimental Section

### Materials

The Cu film was purchased with a thickness of 60 μm. Acetone, ethanol, isopropyl alcohol (IPA), hydrochloric acid (HCl, 37 wt %), sodium chlorite (NaClO_2_), sodium hydroxide (NaOH), and sodium phosphate tribasic dodecahydrate (Na_3_PO_4_·12H_2_O) were all purchased from Sigma-Aldrich, USA. All chemicals were directly used as received without further purification.

### Fabrication of the Cu/CuO Foil-Based Roll

The Cu/CuO film was fabricated as follows: the Cu foil was first cleaned with acetone in an ultrasonic bath for 10 min. After washing it with ethanol, IPA, and DI water in series, the Cu film was dried in a clean argon stream and immediately immersed into a 2.0 m hydrochloric acid solution for 10 min to remove the native oxide film on the surface. Subsequently, the Cu foam was rigorously rinsed with DI water and dried again with a clean argon stream. All of the above operations were carried out at ambient temperature. Next, the alkaline solution composed of NaClO_2_, NaOH, Na_3_PO_4_·12H_2_O, and DI water with a mass percent ratio of 3.75:5:10:100 was heated up to 95 °C, and the cleaned Cu foil was dipped into the alkaline solution for 3 min to form nanostructured CuO. After that, the CuO foil was washed thoroughly with DI water to remove the remaining alkaline solution and dried with an argon stream.

### Materials Characterizations

Scanning electron microscopy (SEM) morphologies were characterized on a Zeiss Supra 25 and the acceleration voltage of the electron gun is 7 kV. The water droplet area was measured by ImageJ. Infrared thermal images of the samples were taken by employing the FLIR A655C thermal camera at a resolution of 640 × 480 with a 25° lens. The contact angle of the samples was measured with a SINDIN SDC-350 contact angle meter. High-speed images were recorded by a Chronos 2.1-HD with a frame rate of 1000 fps.

## Results and Discussion

### Concept of Hydrophilic Roll-Accelerated Water Transportation

**Figure 1:**
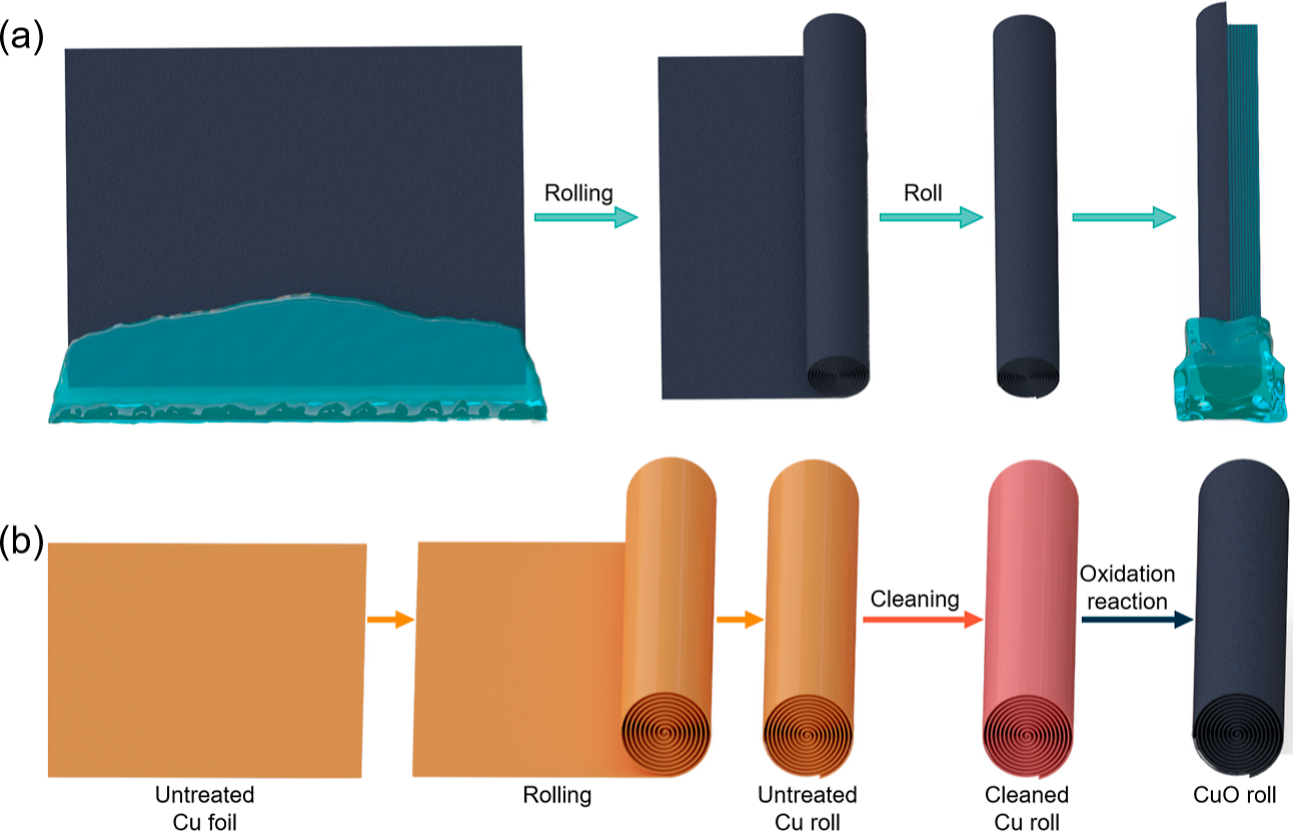
(a) Concept of accelerated water transportation in the Cu/CuO foil-based roll. (b) Fabrication process of the Cu/CuO foil-based roll. The original Cu foil is rolled into a tube and cleaned in series with acetone and hydrochloric acid solution. After a wet-chemical oxidation reaction by immersing the cleaned Cu roll into a hot alkaline solution, the roll turns black with nanostructured CuO growing on the outer surface of the foil. The figures from left to right show the original Cu foil, the rolling process of Cu foil, Cu roll, cleaned Cu roll, and Cu/CuO roll, respectively.

Fast and passive water transportation is favorable in many applications, such as solar-driven evaporation and atmospheric water harvesting. Inspired by the water evaporation/transpiration phenomenon in plants, here, we demonstrate a high-performance and pump-free fabricated from Cu foil using a simple yet effective wet-chemical oxidation method (Figure 1). For the regular Cu/CuO foil, water spread only over a relatively low height above the water surface. After rolling up the Cu/CuO foil, it becomes a 3D roll with a small gap between the layers. The blade-like CuO nanostructure transforms the pristine Cu foil into a superhydrophilic surface. Meanwhile, this unique structure enables enhancement of the capillary effect, which is beneficial for drawing fluids from a bulk reservoir up to the Cu/CuO foil-based roll. The Cu/CuO foil-based roll is fabricated through a facile hot alkaline oxidation process, making it applicable for scale-up employment (Figure 1b). Initially, a planar Cu foil was rolled into a 3D roll and cleaned with organic solvent and hydrochloric acid solution to remove the oil stains and oxidation layer. The cleaned Cu foil showed a shining color. By using a hot alkali solution composed of NaClO_2_, NaOH, Na_3_PO_4_·12H_2_O, and DI water with the chemical solution deposition technique at 95 °C, the nanostructured CuO was fabricated and accompanying the color change from bronze to black. The Cu was oxidized in the chemical processes as follows
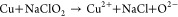

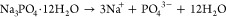

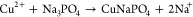

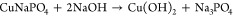

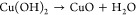


### Materials Characterization and Water Transportation Behavior over a Flat CuO Surface

**Figure 2:**
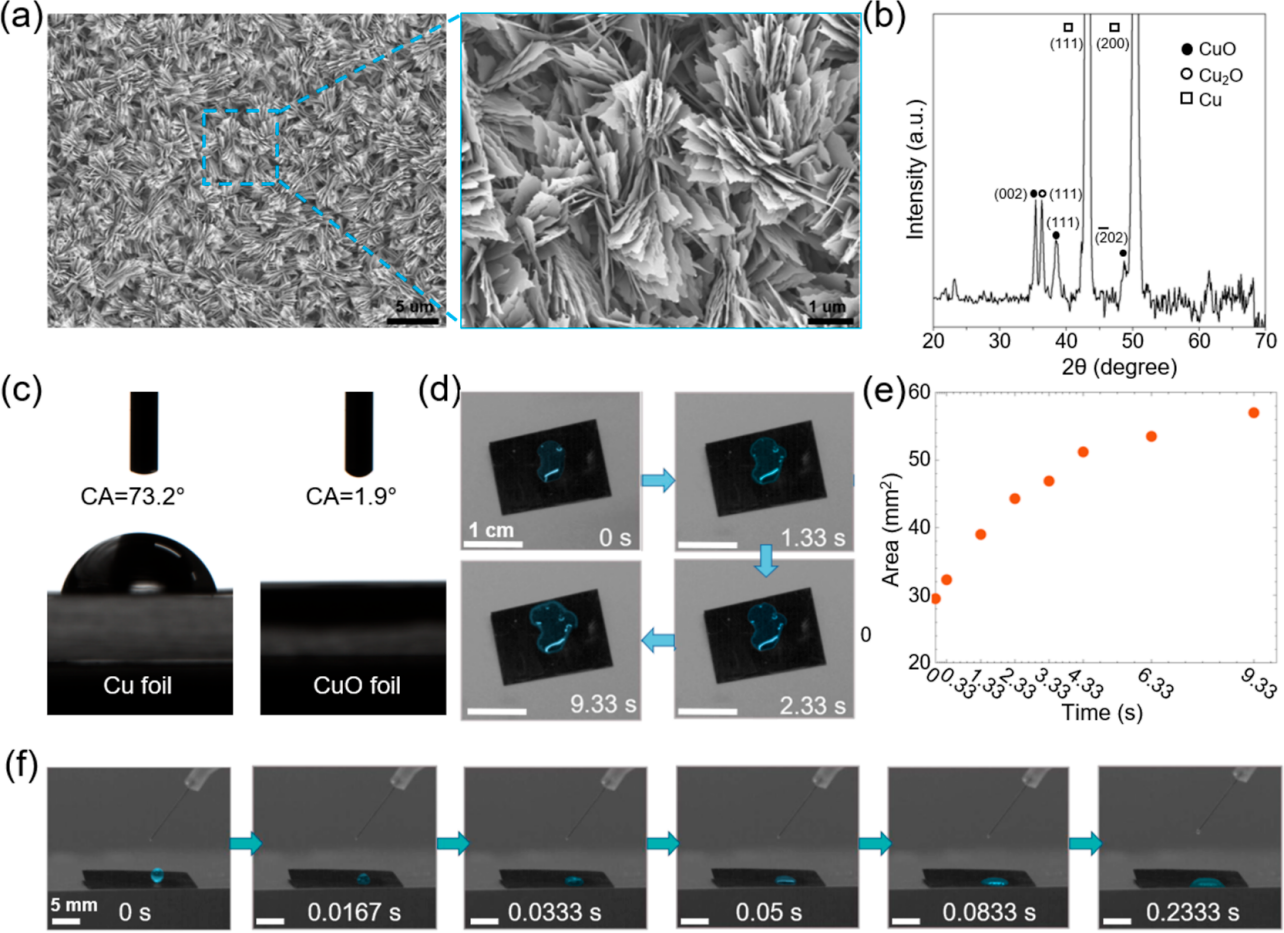
(a) SEM images show the blade-like CuO nanostructures forming on the outer surface of the Cu foil with different magnifications. (b) XRD results of the Cu/CuO foil grown for 1 h. (c) Water contact angles of Cu foil (left) and Cu/CuO foil (right). Time-dependent water diffusion on the Cu/CuO foil in the top view (d) and the corresponding diffusion area (e). Scale bars represent 1 cm. (f) Time-dependent water diffusion on the Cu/CuO foil in the side view. Scale bars represent 5 mm.

SEM images of the Cu/CuO foil are shown in Figure 2a. The nanostructured CuO conforms to the morphology of the Cu foil and the blade-like nanostructured CuO growing on the surface of the Cu foam is shown in the high-magnification SEM images. X-ray diffraction (XRD) measurements are also performed to determine the chemical composition of the oxide layer grown in the alkali solution for 1 h (Figure 2b). The peaks of the XRD spectrum at 35.5, 38.6, and 48.7° are consistent with the standard XRD pattern of the CuO powder. Note that the peaks at values of 43.3 and 50.4° are due to the Cu substrate. After treatment, the contact angle of CuO foil is 1.955° compared with that of the pristine Cu foil, which enables excellent hydrophilicity with rapid water spread over the surface of the Cu foil (Figure 2c). The hydrophilicity of CuO foil was also featured with the quick spread of a water droplet over its top surface (Figure 2d). From 0 to 6.33 s, the area of the water droplet reached 90% of its final area after 9.33 s, according to the area measurement of the water droplet from the top view (Figure 2e). This was also demonstrated by the time-dependent water diffusion images from the side view (Figure 2f). Within 0.2333 s, the droplet has transformed from a sphere to an oval shape, meaning its diffusion over the CuO foil surface is quick.

### Water Transportation Behavior for a Hydrophilic Roll with Different Geometric Configurations

**Figure 3:**
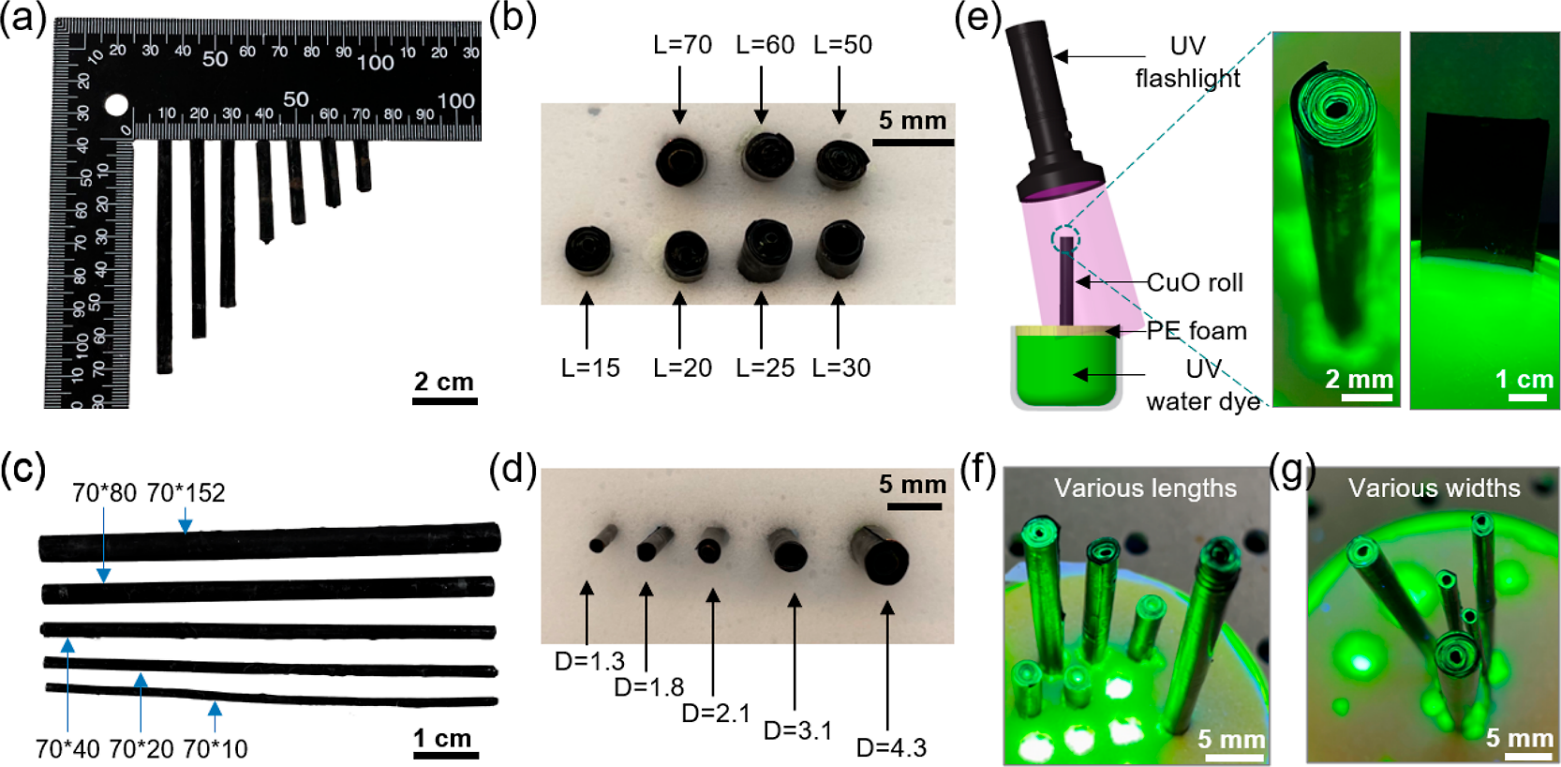
Photos show the Cu/CuO tubes in the longitudinal view (a) and transverse view (b) rolled from foils with different lengths from 15 to 70 mm and a fixed width of 80 mm. The annotation in (b) represents the lengths of the tubes in the mm unit. The photos show the Cu/CuO tubes in the longitudinal view (c) and transverse view (d) rolled from foils with a fixed length of 70 mm and different widths from 10 to 152 mm. The annotation in (d) represents the diameters of the tubes in the mm unit. (e) Schematic showing the experimental setup of water diffusion in the Cu/CuO roll (photo on the left) and the Cu/CuO foil (photo on the right). Photos of Cu/CuO rolls filled with green UV-reactive water in different lengths (f) and different layers (g).

We visually investigated the fluidic transport behavior of the Cu/CuO foil-based roll with different geometric parameters by analyzing the time-dependent climbing height of water with a UV-reactive solution. The Cu/CuO foil-based roll with different lengths (15, 20, 25, 30, 50, 60, and 70 mm) and various diameters (1.3, 1.8, 2.1, 3.1, and 4.3 mm) is fabricated for investigation (Figure 3a–d). Varying diameters mean different widths of the pristine Cu/CuO foils. Figure 3e–g displays the schematic setup for the water transport test. The Cu/CuO rolls are inserted vertically into a beaker filled with water and UV-reactive dye. Under the illumination of UV light, the dye fluoresces a bright yellow-green color used for visual contrast. The length inserted into the dyed water was kept at a fixed length of 10 mm, and the rolls were fixed vertically by inserting them into holes with diameters a little smaller than those of the rolls. Figure 3f,g shows the top-view photos of the Cu/CuO rolls with different lengths and diameters, respectively.

**Figure 4:**
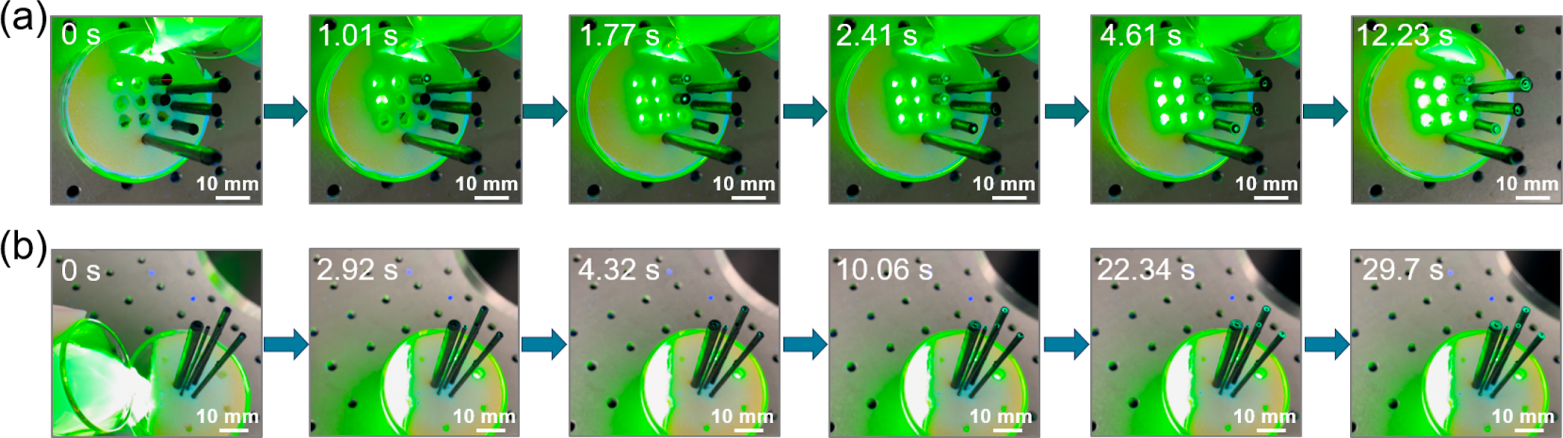
Photos showing time-dependent water diffusion in the Cu/CuO rolls with different lengths (a) and different diameters (b).

Figure 4 shows the time-dependent water transport rate in the test. Due to the strong capillary effect, the water can climb to the end point of each roll. Therefore, the time required to reach the end point can reflect the water transport velocity for rolls with different lengths. Compared with the limited water diffusion height, those rolls can upwardly draw the fluidic solution to a height of 70 mm against fluidic gravity. The spontaneous fluid transport performance of the Cu/CuO rolls is substantially improved. According to the time required for reaching the end point of these rolls, the water diffusion velocity for rolls decreases from 14.9 to 5.7 mm s^–1^ when the length increases from 15 mm to 70 mm, respectively (Figure 4a). For the Cu/CuO rolls with different diameters, the water diffusion velocity decreases from 24.0 to 2.4 mm s^–1^ when the diameter increases from 1.3 to 4.3 mm, respectively (Figure 4b). The efficient upward transport of the fluid in the Cu/CuO rolls originates from its uniquely spiral structure and super hydrophilicity of the CuO surface, in which the small gap between layers of CuO foil mainly helps to draw the fluidic solution due to the capillary effect.

**Figure 5:**
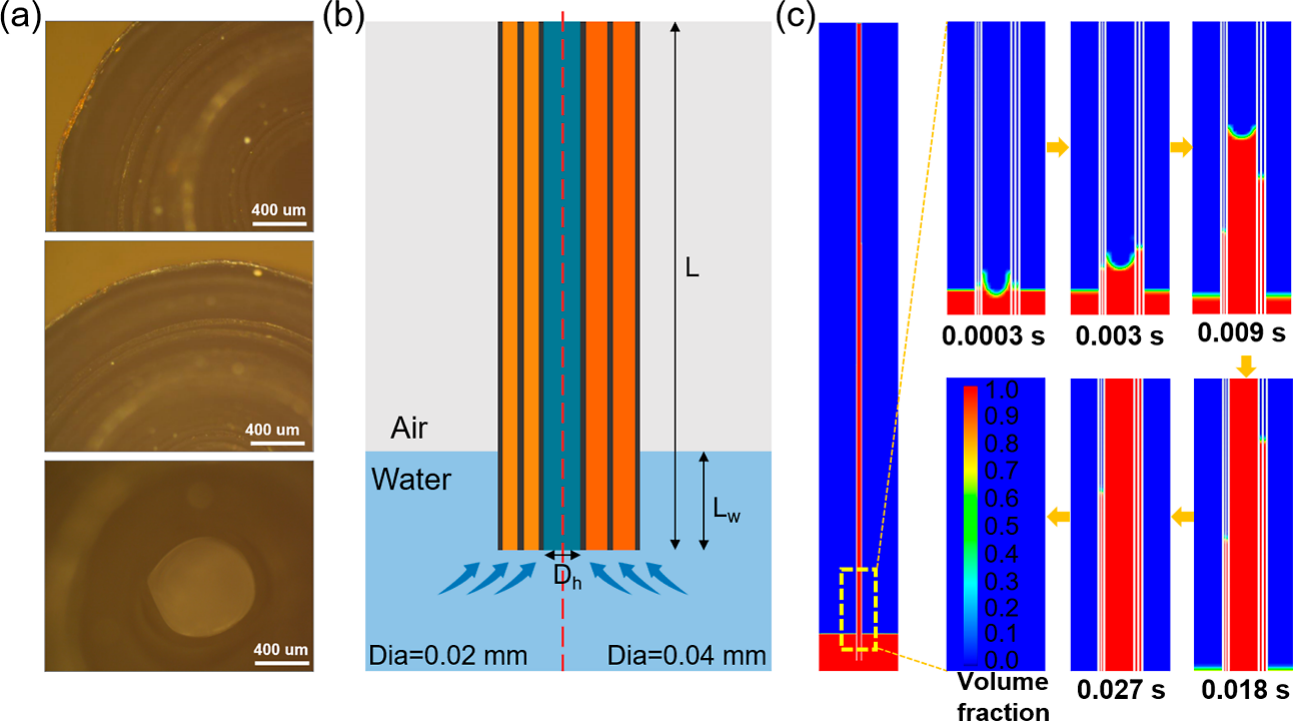
(a) Microscope images showing the top view of the Cu/CuO rolls. (b) Schematic cross-sectional image of the water transportation system showing the fluid domain of the gap in the Cu/CuO roll. (c) Time-dependent volume fraction of water flow distribution in the Cu/CuO roll.

Figure 5a lists the optical microscope images of the top view for Cu/CuO rolls at different magnification factors. Concentric circles are formed with a bigger hollow circle in the center with a diameter of 0.75 mm, and narrow gaps are structured by two adjacent Cu/CuO foils with major dimensions of 0.02 and 0.04 mm. Ansys Fluent is employed to simulate the transient transport phenomenon after Cu/CuO foils are inserted into water. Figure 5b describes the geometric structure used for the Fluent simulation. Details about the simulation were described in the method section. At 0.003 s, the water inside the central hollow circle moves faster than that inside the left and right narrow gaps. At 0.009 s, the water climbing height of the right narrow gap with a diameter of 0.04 mm is higher than the left, which demonstrates that its capillary force is stronger than that of the left part. As time evolves, this difference becomes more clear, i.e., the height difference between the left and right narrow gap becomes larger at 0.018 and 0.027 s.

**Figure 6:**
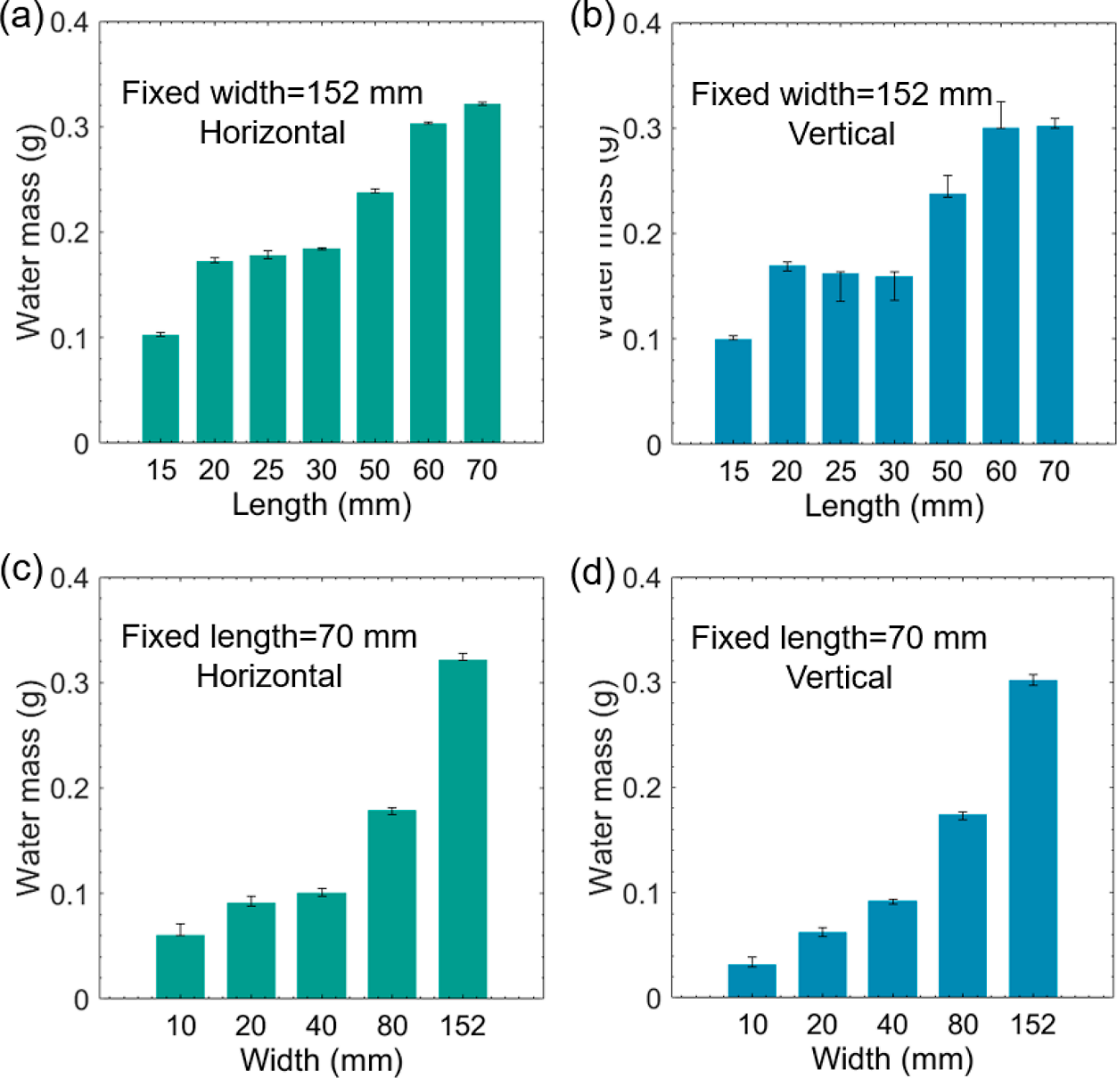
Water uptake of the Cu/CuO rolls in different lengths fully submerged in water (a) and partly inserted into the water vertically (b). Water uptake of the Cu/CuO rolls with different widths was fully submerged in water (c) and partly inserted into the water vertically (d).

We further investigate the water uptake capability of Cu/CuO rolls with different lengths or widths when initially placed horizontally or vertically into the water. The weight of absorbed water is calculated by measuring the mass difference before placing it into the water and after taking it from the water. For a roll with a fixed width of 152 mm that is horizontally placed into water, the weight of absorbed water increases with the increasing of roll length for both the horizontal and vertical scenarios (Figure 6a,b). The water distribution per area is divided by dividing the water mass by the original area of the planar foil. The unit water distribution was almost the same for both horizontal and vertical scenarios. Next, we measured the water uptake capability when the length of the rolls was fixed at 70 mm. The water mass increases with the increasing width for both the horizontal and vertical scenarios (Figure 6c,d). When it is horizontally immersed in water, the unit water distribution for the short width is much higher than that of the vertically immersed rolls. This is because the water mass stored in the central hole accounts for most of the water uptake. Therefore, with the increase of roll diameter, a similar unit water distribution is observed when the width of the pristine CuO foil is above 40 mm.

### Water Evaporation Investigation of the Hydrophilic Roll with Different Geometric Configurations

**Figure 7:**
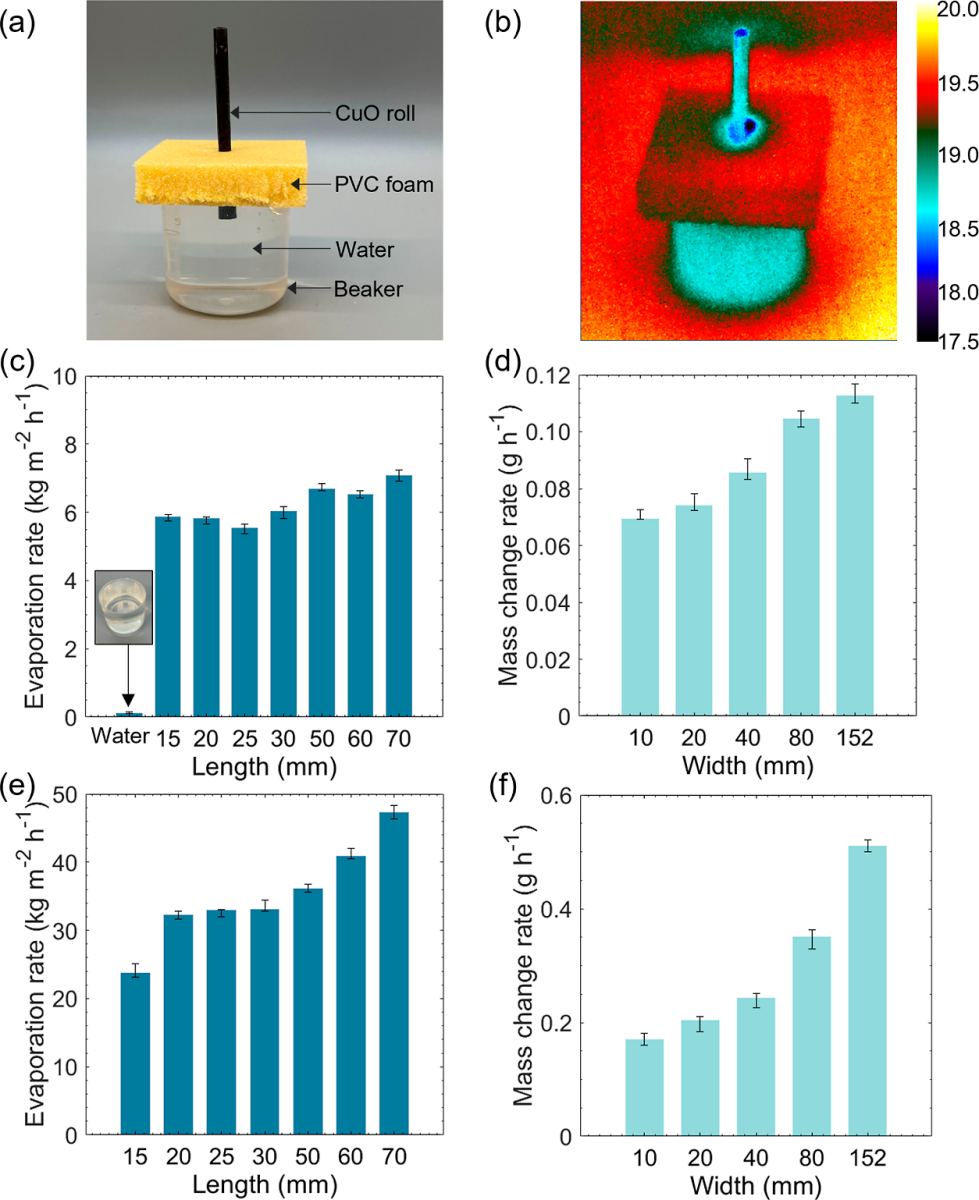
(a) Experimental setup for water evaporation with a Cu/CuO roll in the dark environment. (b) Infrared (IR) thermal image of the Cu/CuO roll in the dark environment with water evaporation. (c) Water evaporation rates with the Cu/CuO rolls in different lengths in the dark environment. (d) Water mass change rates with the Cu/CuO rolls in different widths in the dark environment. (e) Water evaporation rates with the Cu/CuO rolls in different lengths under one sun irradiation. (f) Water mass change rates with the Cu/CuO rolls in different widths under one sun irradiation.

Using this unique design of the hydrophilic roll, we demonstrated an evaporator device that could draw, transport, and evaporate water from the reservoir, as shown in Figure 7a. The Cu/CuO roll was fixed by a PVC foam with a thickness of 10 mm. The length immersed in water is 10 mm. The water evaporation tests in the dark environment lasted over 9.6 h. Figure 7b shows the infrared image of the device when it evaporated in a dark environment. The top circle of the roll has the lowest temperature because of the evaporative cooling effect. We also explored the evaporation rate for the rolls with different geometric parameters (Figure 7c,d). With the increase in roll length, the evaporation rate fluctuates around 6.5 kg m^–2^ h^–1^ since water can climb to the top end and the top surface is the only area for evaporation. For a maximum length of 70 mm, sufficient water can be pumped to the top end for efficient evaporation. The evaporation rate is similar since the area is fixed and the evaporated water mass is similar. Moreover, the evaporation experiments are conducted under one sun (1 kW m^–2^) solar irradiance to examine the water evaporation performance by introducing a solar thermal conversion process since CuO is an excellent solar absorber with approaching unity solar absorptance. As depicted in Figure 7e,f, the water evaporation rate for all Cu/CuO rolls with different structural parameters was significantly augmented due to solar illumination. Under such circumstances, the water evaporation rate increases with the increase of length and width, similar to the result in dark environments. Therefore, the solar thermal conversion process under sunlight can provide a driving force for water transportation and accelerate its water evaporation performance.

## Conclusions

Applications, including solar-driven interfacial evaporation, evaporative cooling, and atmospheric water harvesting, depend on passive water transport by using capillary forces. Passive water movement will be easier through surface engineering and building designs incorporating hydrophilic surfaces and increased capillary force. Here, we present a unique design for a hydrophilic Cu/CuO foil-based roll that speeds up water transit. The metal roll was created by rolling up a standard 2D Cu/CuO film. This changes the behavior of the water when climbing the Cu/CuO film by considerably increasing the capillary force between each adjacent layer. A straightforward spatial translation from a planar foil to a 3D structure has greatly improved water transportation performance and rendered broader practical applications. The Cu/CuO film has an amazing blade-like nanostructure. The nanostructure and its good hydrophilicity ensure the water supply to a specific location, while the water transportation height is increased by the capillary action between the foil’s various layers. The Cu/CuO foil-based roll exhibits a high fluid transport velocity. A wide range of applications, including evaporation in a limited space and evaporation-driven energy harvesting, become possible with this 3D roll design evolved from its 2D planar film.

## References

[ref1] Tao P.; Ni G.; Song C.; Shang W.; Wu J.; Zhu J.; Chen G.; Deng T. Solar-driven interfacial evaporation. Nat. Energy 2018, 3, 1031–1041. 10.1038/s41560-018-0260-7.

[ref2] Tian Y.; Liu X.; Wang Z.; Caratenuto A.; Chen F.; Wan Y.; Zheng Y. Carbonized cattle manure-based photothermal evaporator with hierarchically bimodal pores for solar desalination in high-salinity brines. Desalination 2021, 520, 115345 10.1016/j.desal.2021.115345.

[ref3] Tian Y.; Liu X.; Xu S.; Li J.; Caratenuto A.; Mu Y.; Wang Z.; Chen F.; Yang R.; Liu J.; Minus M. L.; Zheng Y.; Zheng Y. Recyclable and efficient ocean biomass-derived hydrogel photothermal evaporator for thermally-localized solar desalination. Desalination 2022, 523, 115449 10.1016/j.desal.2021.115449.

[ref4] Liu X.; Tian Y.; Chen F.; Caratenuto A.; DeGiorgis J. A.; ELSonbaty M.; Wan Y.; Ahlgren R.; Zheng Y. An easy-to-fabricate 2.5 D evaporator for efficient solar desalination. Adv. Funct. Mater. 2021, 31, 2100911 10.1002/adfm.202100911.

[ref5] Li J.; Jing Y.; Xing G.; Liu M.; Cui Y.; Sun H.; Zhu Z.; Liang W.; Li A. Solar-driven interfacial evaporation for water treatment: advanced research progress and challenges. J. Mater. Chem. A 2022, 10, 18470–18489. 10.1039/d2ta03321f.

[ref6] Tong C. Advanced Materials and Components for 5G and Beyond; Springer, 2022; pp 173–215.

[ref7] Li J.; Wang X.; Liang D.; Xu N.; Zhu B.; Li W.; Yao P.; Jiang Y.; Min X.; Huang Z.; Zhu S.; Fan S.; et al. A tandem radiative/evaporative cooler for weather-insensitive and high-performance daytime passive cooling. Sci. Adv. 2022, 8, eabq0411 10.1126/sciadv.abq0411. 35960798 PMC9374334

[ref8] Chu J.; Huang X. Research status and development trends of evaporative cooling air-conditioning technology in data centers; Energy and Built Environment, 2021.

[ref9] Han Z.; Xue D.; Wei H.; Ji Q.; Sun X.; Li X. Study on operation strategy of evaporative cooling composite air conditioning system in data center. Renewable Energy 2021, 177, 1147–1160. 10.1016/j.renene.2021.06.046.

[ref10] Choi M.; Park S.; Choi W.; Kim Y.; Cho K. M.; Heo J.; Kim M.-K.; Jung H.; Jin Y.; Lee S.; et al. Highly durable and sustainable heterogeneous fabric using in-and-out fluorinated urethane coating for elimination of bacteria and oil–water separation. npj Clean Water 2022, 5, 48 10.1038/s41545-022-00191-0.

[ref11] Yan L.; Yang X.; Li Y.; Song R.; Lin Y.; Huang Q.; Shao L. Acid-resistant supramolecular nanofibrous hydrogel membrane with core-shell structure for highly efficient oil/water separation. J. Membr. Sci. 2023, 679, 121705 10.1016/j.memsci.2023.121705.

[ref12] Yan L.; Yang X.; Zeng H.; Zhao Y.; Li Y.; He X.; Ma J.; Shao L. Nanocomposite hydrogel engineered hierarchical membranes for efficient oil/water separation and heavy metal removal. J. Membr. Sci. 2023, 668, 121243 10.1016/j.memsci.2022.121243.

[ref13] Wen Y.; Yang X.; Li Y.; Yan L.; Zhao Y.; Shao L. Progress reports of metal-phenolic network engineered membranes for water treatment. Sep. Purif. Technol. 2023, 320, 124225 10.1016/j.seppur.2023.124225.

[ref14] Zheng L. J.; Kang H. W. A passive evaporative cooling heat sink method for enhancing low-grade waste heat recovery capacity of thermoelectric generators. Energy Convers. Manage. 2022, 251, 114931 10.1016/j.enconman.2021.114931.

[ref15] Chen W.; Liu S.; Lin J. Analysis on the passive evaporative cooling wall constructed of porous ceramic pipes with water sucking ability. Energy Build. 2015, 86, 541–549. 10.1016/j.enbuild.2014.10.055.

[ref16] Butt H.-J.; Kappl M. Normal capillary forces. Adv. Colloid Interface Sci. 2009, 146, 48–60. 10.1016/j.cis.2008.10.002. 19022419

[ref17] Kralchevsky P. A.; Denkov N. D. Capillary forces and structuring in layers of colloid particles. Curr. Opin. Colloid Interface Sci. 2001, 6, 383–401. 10.1016/S1359-0294(01)00105-4.

[ref18] Lambert P.; Chau A.; Delchambre A.; Régnier S. Comparison between two capillary forces models. Langmuir 2008, 24, 3157–3163. 10.1021/la7036444. 18315017

[ref19] Bai H.; Wang X.; Li Z.; Wen H.; Yang Y.; Li M.; Cao M. Improved Liquid Collection on a Dual-Asymmetric Superhydrophilic Origami. Adv. Mater. 2023, 35, 2211596 10.1002/adma.202211596. 36807414

[ref20] Miljkovic N.; Enright R.; Nam Y.; Lopez K.; Dou N.; Sack J.; Wang E. N. Jumping-droplet-enhanced condensation on scalable superhydrophobic nanostructured surfaces. Nano Lett. 2013, 13, 179–187. 10.1021/nl303835d. 23190055

